# Seroprevalence of Hepatitis-E Virus-Immunoglobulin G and its association with Chronic Liver Disease

**DOI:** 10.12669/pjms.40.5.8448

**Published:** 2024

**Authors:** Muhammad Sadik Memon, Bushra Kadir, Lubna Kamani, Ayaz Ahmed Chandio

**Affiliations:** 1 Muhammad Sadik Memon, Professor, Asian Institute of Medical Sciences (AIMS) Hospital, Hyderabad, Pakistan; 2 Bushra Kadir, Consultant Gastroenterologist, Asian Institute of Medical Sciences (AIMS) Hospital, Hyderabad, Pakistan; 3 Lubna Kamani, Professor & Director GI Residency Program, Liaquat National Hospital, Karachi, Pakistan; 4 Ayaz Ahmed Chandio, Biostatician, Asian Institute of Medical Sciences (AIMS) Hospital, Hyderabad, Pakistan

**Keywords:** Hepatitis, HEV, Chronic liver disease

## Abstract

**Background & Objective:**

Viral hepatitis is a major public health concern in low-middle income countries. Hepatitis-E infection (HEV) is found globally but most prevalent in low-income countries especially those with poor sanitation systems, access to clean drinking water and health services. Superinfection with HEV in patients with chronic liver disease (CLD) can cause severe hepatic decompensation leading to increased morbidity and mortality. To determine the frequency of seroprevalence of Hepatitis-E virus Immunoglobulin g (IgG) and its association with chronic liver disease.

**Methods:**

A cross-sectional study was conducted in Asian Institute of Medical Sciences, Hyderabad, Pakistan from January till May 2022. A total of 196 patients of aged ≥ 18 years, presenting in gastroenterology clinics were included in the study after informed consent.

**Result:**

Among 196 patients, one third of patient were male (73.5%). Out of which 162 (82.7%) had liver disease and 34 (17.3%) were without liver disease. The median age of patient was 45 (33-51) years. The overall seroprevalence of HEV IgG among study population was 69.4%. HEV IgG was present in 114 and 22 in CLD and non CLD patients respectively. Multivariable regression shows no association between seroprevalence of HEV in CLD and non-CLD patient (AOR 1.02, 95% CI 0.45-2.313).

**Conclusion:**

Our study showed high frequency of HEV seropositivity. No difference was observed in HEV seropositivity among CLD and non-CLD patients.

## INTRODUCTION

Hepatitis-E virus (HEV) is a single-stranded RNA enveloped virus discovered in 1983. The most common route of transmission of HEV is fecal-oral route.^1^ Patient infected with HEV may develop acute or sub-clinical hepatitis. However, in developing countries, HEV infections are endemic, and it may spread with or without any history of traveling to endemic areas.^2^-^4^ In South America, Africa and Asia, HEV is considered as the most common etiology of water borne hepatitis.^5^,^6^ According to WHO, the estimated annual burden of Hep E infection is twenty million globally. It remains a global problem, mostly commonly occurring in South and East Asia. In 2015 HEV was responsible for 44,000 deaths.^7^ HEV infection is found globally but most prevalent in low-income countries especially those with poor sanitation systems, access to health services and clean drinking water. In slum area, the disease occurs as sporadic cases as well as outbreaks. Few of these outbreaks have occurred in areas of humanitarian emergencies and camps for refugees or internally displaced populations with poor sanitation conditions. It is usually a self-limiting illness, but can lead to fulminant hepatic failure in elderly, immune-compromised patients and pregnant females.^7^

Studies shows that superinfection of HEV can leads to severe illness in patient with CLD. In endemic countries majority of the CLD patients are prone to get HEV infection and are ideal candidates for an HEV vaccine.^8^ On the other hand, the seroprevalence data of HEV shows that about two billion people have been estimated to be infected by HEV globally in general population.^9^ Given the context of the burden of HEV among general population, and CLD patients in endemic areas like South Asia and Pakistan, there has been a need to evaluate the seroprevalence of HEV among CLD and non-CLD patients. A comparison will help in better understanding of the relation between HEV exposure and CLD on the basis of scientific evidence, its severity and the need of vaccination against HEV. This study aimed to evaluate the impact of previous exposure of HEV in CLD and controlled population, its impact of aggravating the CLD and its seroprevalence, as well as need of vaccination against it in CLD patients.

## METHODS

A cross-sectional study was conducted in Asian institute of medical sciences, Hyderabad, from January till May 2022. The study was carried out with the approval of the hospital ethical committee (AIMS/ERC/7738/16-05-22). The process of sample collection was done randomly, Applying Simple Random sampling technique, Patients were enrolled from gastroenterology out-patient department (OPD) after obtaining the informed consent. Patients of aged ≥ 18 years, either gender, presenting with any complaint in gastroenterology OPD were enrolled in the study. However, patients with malignancy and chronic kidney disease were excluded.

The sample size was determined by using the WHO sample size calculator. Previous study had reported the frequency of HEV IgG positive to be 26.7% in patients with chronic liver disease.^10^ the estimated sample size was 196 patients in this study with a 95% confidence interval and a 6.2% margin of error. Chronic liver disease is defined as patient having any type of chronic liver disease such as HBV, HCV, cryptogenic or non-alcoholic steatohepatitis for at least six months. Laboratory investigations included alpha fetoprotein, albumin, liver function test and platelets, prothrombin time, ultrasound abdomen. Whereas, Patients with Negative virology (B, C) and normal ultrasound, no history of liver disease or diagnosis in past were labelled as Non CLD.

Hepatitis-E immunoglobulin G was performed by ELISA method. The designated data collector recorded the patient’s demographic details such as age, gender, and clinical history, and comorbid, history of upper gastrointestinal bleeding (UGIB), jaundice, ascites, chronic liver disease and results of laboratory investigation.

All the collected data was entered and analyzed by using SPSS version 26. Median (IQR) was reported for quantitative variables whereas frequencies and percentages were calculated for categorical variables. Impact of HEV infection on chronic liver disease will be evaluated by using binary regression analysis. Firstly, univariate analysis was performed and all variables having p-value ≤ 0.25 included in multivariable model. However, in multivariable model, variable will be retained in model based on clinical significance or p-value < 0.1. Adjusted and unadjusted odds ratio with 95% CI was calculated considering p-value ≤ 0.05 as significant.

## RESULTS

In this study we enrolled 196 patients of age ≥18 years. Out of which 162 (82.7%) had liver disease and 34 (17.3%) were without liver disease. The median age of patient was 45 (33-51) years. Among 196 patients, 144 (73.5%) were males with male to female ratio around 3:1. However, the median total bilirubin of the study population was 0.8 mg/dl with ALT, AST, albumin, PT and AFP of 35, 34,5, 3.4, 12.0 and 4.0 respectively.

**Fig.1:**
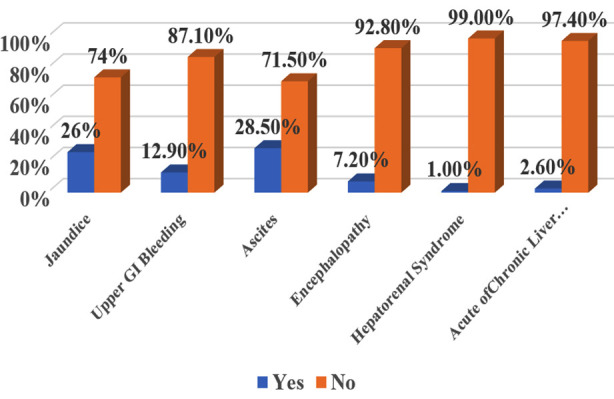
Distribution of history of jaundice, UGIB, ascites, encephalopathy, HRS and ACLF among study population

**Fig.2:**
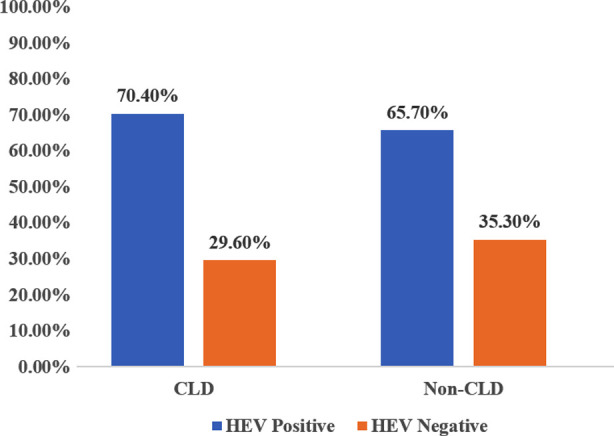
Seroprevalence of HEV IgG among CLD and non-CLD patient.

Furthermore, history of jaundice was reported in 26%, upper GI bleeding in 12.9%, ascites in 28.5% and encephalopathy in 7.2%. Details are shown in Fig1. In addition, the most common comorbid among the study population was obesity (19.4%) followed by diabetes (7.1%) and hypertension (2.6%). The overall seroprevalence of HEV IgG among study population was 69.4%. However, seroprevalence of HEV is slightly higher in CLD patient as compared to non-CLD patient as shown in Fig.2.

The association of difference variables with HEV IgG in terms of crude and adjusted odds ratio is shown in Table-I. There was only one variable, i.e., total bilirubin, found to be significant in multivariable binary regression analysis. Odds of having positive HEV IgG among patients with raised total bilirubin was 5.27 times higher as compared to normal total bilirubin.

**Table-I:** Univariate and Multivariate Binary regression analysis.

	HEV IgG			

Predictor	No	Yes	COR (95% CI)	AOR (95% CI)
***Age***				
≤ 45	40 (35.4)	73 (64.6)	Ref	
> 45	20 (24.1)	63 (75.9)	1.726 (0.916-3.253)	1.627 (0.838-3.16)
***Gender***				
Male	48 (33.3)	96 (66.7)	Ref	
Female	12 (23.1)	40 (76.9)	1.667 (0.801-3.466)	1.876 (0.889-3.962)
***CLD***				
No	12 (35.3)	22 (64.7)	Ref	
Yes	48 (29.6)	114 (70.4)	1.295 (0.594-2.826)	1.02 (0.45-2.313)
***AFP***				
Normal	49 (31.6)	106 (68.4)	Ref	
Abnormal	11 (26.8)	30 (73.2)	1.261 (0.584-2.721)	
***Total Bilirubin***				
Normal	58 (33.5)	115 (66.5)	Ref	
Raised	2 (8.7)	21 (91.3)	5.296 (1.2-23.365)	5.427 (1.208-24.374)
***Platelet Count***				
Normal	37 (34.9)	69 (65.1)	Ref	
Thrombocytopenia	21 (23.9)	67 (76.1)	1.711 (0.909-3.22)	
Thrombocytosis	2 (100)	0 (0)	--	
***ALT***				
Normal	46 (32.4)	96 (67.6)	Ref	
Raised	14 (25.9)	40 (74.1)	1.369 (0.678-2.765)	
***AST***				
Normal	40 (29.6)	95 (70.4)	Ref	
Raised	20 (33.9)	39 (66.1)	0.821 (0.427-1.578)	
***Hypoalbuminemia***				
No	35 (35)	65 (65)	Ref	
Yes	25 (26)	71 (74)	1.529 (0.828-2.825)	
***H/o Jaundice***				
No	47 (32.4)	98 (67.6)	Ref	
Yes	13 (25.5)	38 (74.5)	1.402 (0.683-2.878)	
***H/o UGIB***				
No	54 (32)	115 (68)	Ref	
Yes	5 (20)	20 (80)	1.878 (0.669-5.271)	
***H/o Ascites***				
No	47 (34.1)	91 (65.9)	Ref	
Yes	11 (20)	44 (80)	2.066 (0.977-4.368)	
***CTP Class***				
A	44 (36.1)	78 (63.9)	Ref	
B	15 (23.4)	49 (76.6)	1.843 (0.928-3.66)	
C	1 (10)	9 (90)	5.077 (0.622-41.408)	
***Herbal Treatment***				
No	60 (30.6)	136 (69.4)	--	
Yes	0	0		

HEV: Hepatitis-E Virus, IGG: Immunoglobulin G, CLD: Chronic Liver Disease, AFP: Alpha Fetoprotein, ALT: Alanine Transaminase, ALT: Aspartate Aminotransferase, UGIB: Upper gastrointestinal bleeding, CTP: Child-Turcotte-Pugh, COR: Crude odds ratio, AOR: Adjusted odds ratio, CI: confidence interval.

## DISCUSSION

In this study we enrolled 196 patients of age ≥18 years. The median age of patient was 45 (33-51) years and 73.5% patients were male in our study. Moreover, which 162 (82.7%) had liver disease and 34 (17.3%) were without liver disease. The median total bilirubin of the study population was 0.8 mg/dl with ALT, AST, albumin, PT and AFP of 35, 34,5, 3.4, 12.0 and 4.0 respectively. The overall seroprevalence of HEV IgG among study population was 69.4%. However, seroprevalence of HEV is slightly higher in CLD patient as compared to non-CLD patient. Viral hepatitis is a major public health concern in developing countries like Pakistan, India and Bangladesh. In developed countries like those in Europe and the USA, occasionally, cases have been reported.^11^,^12^ Transmission of HEV through blood has also been reported because some studies show higher prevalence of HEV among blood donors.^13^-^15^ HEV infection can rapidly progress and leads to liver failure in patients with organ transplant, malignancy and those with coexisting HIV infection.^16^ Additionally, HEV has produced negative effects on pregnant women, resulting in prenatal mortality rates of 30.3 per 1000 live births and maternal mortality rate ranging between 20 and 29.3%.^17^

In several Asian countries HEV is considered as an endemic.^18^ Nevertheless, most of the cases in China and India are due to genotype 4 of HEV, while in Japan most of the cases are due to genotypes three and four.^19^ In Pakistan, first report assessed association of HEV infection with liver decompensation.^20^ In that study, 233 patient with CLD were screened for viral hepatitis (HAV and HEV). Surprisingly, HEV superinfection was reported in 100% of patient with liver decompensation and CLD. HEV superinfection in patients with CLD may aggravate the course of underlying conditions.^21^,^22^ Furthermore, HEV infection is associated with mortality in patients with pre-existing CLD.^23^

In our study the overall seroprevalence of Hepatitis-E infection was 69.4%. Previous studies conducted in Brazil and Pakistan showed a lower prevalence of HEV infection in general population as well as in patient with chronic liver disease.^23^-^26^ Furthermore, the prevalence of Hepatitis-E infection was also affected by geographical location. Therefore, a large variation was seen in HEV prevalence among the general population such as 50-100% in Egypt, 39-42% in USA, 22.5% in Bangladesh and 1.9% in Netherlands.^2^,^27^,^28^

Similarly, seroprevalence of HEV infection was slightly higher in CLD patients as compared to non-CLD. Study conducted in 2012-2013 by Hoan et al reported seroprevalence of HEV in CLD and non-CLD patient was 45% and 31% respectively.^29^ Another study from Turkey observed prevalence of HEV infection in HBV and HCV patient was 13.7% and 54% respectively.^30^ One recent study enrolled 600 patients with CLD observed seroprevalence of HEV infection 28.5%.^31^

Our study found that there was no difference in seroprevalence of HEV infection among either gender. These findings are consistent with existing literature which shows equal HEV infection rate in both genders.^25^ However, some studies have reported higher seroprevalence of HEV infection among males.^32^ This discrepancy in results may be due to unequal distribution of males and females in this study. Moreover, in developing countries females have limited treatment opportunities. However, in the current study, no association was found between age and seroprevalence while previous studies have shown that HEV infection increased with advancing age.^33^

In another previous study, the HEV seroprevalence among younger patients was 14.3% compared to elderly patients which increased up to 90.9%.^34^ However, in Pakistani populations studies on seroprevalence and its association with CLD are scarce. Findings of our study will help in bridging the existing knowledge regarding epidemiology of HEV infection and its impact on CLD. In addition, understanding HEV co-infection burden in CLD patients is clinically important because existing literature demonstrate that co-infection of HEV is associated with a more aggressive clinical course, often leading to acute decompensation and unfavorable outcomes.^29^,^30^,^35^

### Limitations

Although our study presents significant data that emphasizes the epidemiology of HEV infection, particularly among individuals with CLD, it is important to recognize certain limitations when interpreting the findings of our study. Firstly, it’s a single center study, center is also a liver patient hospital, with small sample size, so the findings of our study may not be generalized for overall population. Finally, the data regarding important risk factors such as lifestyle, dietary habits and family history was not collected. However, in spite of this limitation, this study is the first one which evaluated the association of HEV IgG with chronic liver disease.

For control of HEV infection there is need to create awareness regarding hygienic conditions. Public health professionals, policy makers and clinicians should work together to develop strategies to improve the hygienic conditions of people living in Karachi. Furthermore, large population-based studies are needed to confirm the seropositivity of HEV infection and its association with chronic liver disease.

## CONCLUSION

Our study observes overall high frequency of HEV virus. However, there is no difference was observed in HEV infection among CLD and non-CLD patients. Findings of our study highlights the necessity to enhance HEV diagnostic testing, as well as reevaluate the potential role of HEV vaccines in CLD patients who are at a higher risk of decompensation and mortality due to acute HEV infection.

### Authors’ Contribution:

**MSM:** Conceptualization, protocol writing, integrity and accountability of work.

**BK:** Data collection and manuscript revision.

**LK:** Manuscript writing and editing.

**AAC:** Data analysing and writing writing/compiling final results.
